# Evolution of Heterogeneity (I^2^) Estimates and Their 95% Confidence Intervals in Large Meta-Analyses

**DOI:** 10.1371/journal.pone.0039471

**Published:** 2012-07-25

**Authors:** Kristian Thorlund, Georgina Imberger, Bradley C. Johnston, Michael Walsh, Tahany Awad, Lehana Thabane, Christian Gluud, P. J. Devereaux, Jørn Wetterslev

**Affiliations:** 1 Department of Clinical Epidemiology and Biostatistics, McMaster University, Hamilton, Ontario, Canada; 2 Copenhagen Trial Unit, Department 3344, Centre for Clinical Intervention Research, Rigshospitalet, Copenhagen University Hospital, Copenhagen, Denmark; 3 Department of Anaesthesia and Pain Medicine, The Hospital for Sick Children, Toronto, Ontario, Canada; 4 Division of Nephrology, Department of Medicine, McMaster University, Hamilton, Ontario, Canada; 5 Biostatistics Unit, Father Sean O'Sullivan Research Centre, St Joseph's Healthcare – Hamilton, Hamilton, Ontario, Canada; 6 Public Health Research Institute, McMaster University, Hamilton, Ontario, Canada; Queen's University Belfast, United Kingdom

## Abstract

**Background:**

Assessment of heterogeneity is essential in systematic reviews and meta-analyses of clinical trials. The most commonly used heterogeneity measure, *I^2^*, provides an estimate of the proportion of variability in a meta-analysis that is explained by differences between the included trials rather than by sampling error. Recent studies have raised concerns about the reliability of *I^2^* estimates, due to their dependence on the precision of included trials and time-dependent biases. Authors have also advocated use of 95% confidence intervals (CIs) to express the uncertainty associated with *I^2^* estimates. However, no previous studies have explored how many trials and events are required to ensure stable and reliable *I^2^* estimates, or how 95% CIs perform as evidence accumulates.

**Methodology/Principal Findings:**

To assess the stability and reliability of *I^2^* estimates and their 95% CIs, in relation to the cumulative number of trials and events in meta-analysis, we looked at 16 large Cochrane meta-analyses - each including a sufficient number of trials and events to reliably estimate *I^2^* - and monitored the *I^2^* estimates and their 95% CIs for each year of publication. In 10 of the 16 meta-analyses, the *I^2^* estimates fluctuated more than 40% over time. The median number of events and trials required before the cumulative *I^2^* estimates stayed within +/−20% of the final *I^2^* estimate was 467 and 11. No major fluctuations were observed after 500 events and 14 trials. The 95% confidence intervals provided good coverage over time.

**Conclusions/Significance:**

*I^2^* estimates need to be interpreted with caution when the meta-analysis only includes a limited number of events or trials. Confidence intervals for *I^2^* estimates provide good coverage as evidence accumulates, and are thus valuable for reflecting the uncertainty associated with estimating *I^2^*.

## Introduction

Measures of heterogeneity are essential in systematic reviews and meta-analyses of clinical trials [1]–[6]. The most commonly used heterogeneity measure, *I^2^*, provides an estimate of the proportion of variability in a meta-analysis that is explained by differences between the included trials rather than by sampling error [2], [3]. Several studies have demonstrated important shortcomings of the *I^2^* measure [7]–[12]. *I^2^* estimates may be particularly unreliable in meta-analyses including a small number of trials (e.g., less than 10 trials) due to lack of power [7], [8]. *I^2^* estimates may be underestimated as a result of time-lag bias [9], [10]. Moreover, comparably higher or lower precision in the most recently added trials may inflate or deflate *I^2^* under different circumstances [8], [11].

Imprecise or biased estimates of heterogeneity can have serious consequences [6], [12]. Underestimation of heterogeneity may inappropriately prevent exploration of the cause(s) of heterogeneity. Overestimation of heterogeneity may inappropriately prevent a meta-analysis actually being done. Overestimation may also trigger inappropriate exploration of the cause(s) of heterogeneity. For example, large I^2^ estimates may prompt authors to exhaust all possibilities of subgroup analyses – a conduct notorious for its tendency to yield findings beyond replication [13].

In response to the above identified shortcomings, it has been proposed that reported *I^2^* estimates should be accompanied by their associated 95% confidence interval (CI) [6], [12]. Confidence intervals may be a desirable addition to the single I^2^ estimate; they give an appreciation of the spectrum of possible degrees of heterogeneity (e.g., mild to moderate), allowing for more appropriate interpretation of the overall intervention effect estimate. One concern, however, is the possibility that the I^2^ estimate's dependence on power, trial weights, and time-lag bias may cause fluctuations beyond the play of chance. With such fluctuations, the 95% CIs may not retain their desired coverage.

To explore these issues we selected a sample of 16 large Cochrane meta-analyses, each including a sufficient number of trials, patients and events to provide reliable treatment effect estimates and I^2^ estimates. We retrospectively re-analysed the data for each meta-analysis, starting with the first chronological trial, and calculating a cumulative I^2^ estimate and its associated 95% CI after each new trial was added to the meta-analysis. We then estimated the number of events and trials generally needed for I^2^ estimates and 95% CIs to converge.

### Statistical framework and theoretical considerations

In this section we first outline the construct of the *I^2^* measure and its associated 95% CI. We secondly provide an overview of meta-analysis factors and properties of the *I^2^* measure that may inappropriately affect the magnitude of the *I^2^* estimate. Lastly, we provide the rationale for empirically studying *I^2^* estimate and their associated 95% CIs over time.

### Measuring heterogeneity between studies

Higgins et al. proposed the now widely popular measure of heterogeneity, *I^2^*, as well as methods for calculating the associated 95% CIs [2], [3]. *I^2^* expresses the proportion of variability in a meta-analysis which is explained by between-trial heterogeneity rather than by sampling error. Mathematically, *I^2^* is expressed as *I^2^ = τ^2^/(σ^2^+τ^2^)*, where *τ^2^* denotes the between-trial heterogeneity, *σ^2^* denotes some common sampling error across trials, and *σ^2^+τ^2^* is the total variation in the meta-analysis. *I^2^* is usually calculated as (*Q−df)/Q×100%*, where *Q* is the Cochran's homogeneity test statistic and *df* is the degrees of freedom (the number of trials minus 1) [2], [3], [14]. Higgins et al. explored a number of methods for obtaining 95% CIs of the *I^2^* estimate [2]. For this study, we will use the method referred to as the *test based* method in Higgins et al. [2] This method yields good coverage in most situations and is easy to calculate [2]. The required calculations for this method are outlined in the appendix S1.

### Factors affecting I^2^ estimates

*I^2^* estimates may be unreliable due to lack of power and precision [7], [8], [11], due to the presence of time-dependent biases [9], [10], or due to dependence on trial weights and precisions.

#### Power and precision

Since *I^2^* is a monotonically increasing function of Cochran's *Q*, large values for *Q* result in large *I^2^* estimates and small values for *Q* result in small *I^2^* estimates. The power of Cochran's *Q* depends on the number of trials and the precision of the trials (i.e., the number of patients and events in the trials) [7], [8], [11]. When the number of trials or their respective precision are small, Cochran's Q usually has inappropriately low power to detect heterogeneity, and therefore tends to yield conservative (low) test values [7], [8]. To illustrate, the median number of trials is seven for Cochrane meta-analyses and 12 for meta-analyses published in paper journals [15], [16]. The median sample size in randomized clinical trials is typically less than 100 in most medical specialties [17], [18]. Thus, it is common for Cochran's Q to have low power. This lack of power is likely to cause underestimation of *I^2^*, particularly if there a few events among the included trials [7].

#### Time-dependent bias

Time-dependent bias (i.e., time-lag bias and publication bias) is known as a threat to the validity of the pooled estimate of effect in meta-analyses [19]–[21]. In addition, time-dependent bias may compromise the validity of heterogeneity estimates [9], [10]. It is accepted that statistically significant trials with large intervention effect estimates usually get published the fastest [21]. If a meta-analysis is conducted at a time where all trials yield large promising treatment effects, the similarity across trials will result is a relatively small *I^2^* estimate. If the meta-analysis is updated some years later, this update is likely to include trials that found more moderate, neutral, or negative treatment effects. The inclusion of such trials will generate larger estimates of heterogeneity.

#### Dependence on trial weights and precisions

From the mathematical expression *I^2^ = τ^2^/(σ^2^+τ^2^)*, it is clear that relatively large sampling errors across trials will result in small *I^2^* estimates, and conversely, that relatively small sampling errors across trials will result in large *I^2^* estimates [2], [3], [8], [11]. The “common” sampling error, *σ^2^*, across trials may change considerably over time. For example, if early trials enroll a more homogeneous or heterogeneous set of patients than later trials, if they have shorter follow-up than later trials, or if changes are made to the definition of the outcome measure (e.g., the definition of myocardial infarction has changed considerably over the past decades); then the “common” sampling error may be considerably different at a later stage in a meta-analysis than it was in the early stage.

Provided the between study variance, *τ^2^*, remains relatively stable over time, changes in the “common” sampling error may cause considerable changes in *I^2^* estimates over time. Further, if the between-study variance incurs considerable changes over time, changes in the “common” sampling error may either inflate or deflate the representation of such changes through the *I^2^* estimate.

### The need to assess convergence of I^2^ estimates and confidence intervals

From the above discussion, it is evident that *I^2^* estimates may incur considerable fluctuations over time. Currently, no studies have explored the magnitude of this problem, and no recommendations exist as to how many events or trials are needed to achieve adequately stable *I^2^* estimates in meta-analysis.

It has been proposed that *I^2^* should be reported with their associated 95% CIs. By construct, the conventional frequentist CI represents the spectrum of results that would include the true underlying value in a particular proportion (typically 95%) if the experiment were independently repeated many times. In meta-analysis, we can conceptually think of an ‘experiment’ as a set of trials ‘sampled’ randomly from a universe of all possible trials. However, as outlined in the above sections, the patterns with which different types of trials are included in a meta-analysis over time are typically not random. For example, small trials are likely to precede larger trials. Thus, the statistical assumptions on which *I^2^* confidence intervals are based may not hold in many meta-analyses. For this reason, it is important to explore, empirically, how 95% confidence intervals perform as more trials are accumulated over time.

## Materials

In a previous empirical study, we extracted data from 920 binary ‘primary outcome’ meta-analyses in Cochrane systematic reviews [22]. We had defined primary outcomes as one of the first three outcomes in the first comparison group [22]. The data set only included meta-analyses that pooled results across all trials; meta-analyses reporting only sub-totals were excluded. For this current study, we used the same population of meta-analyses and selected the subset of meta-analyses that met the following eligibility criteria:

The total number of included trials surpassed 30. We employed this eligibility criterion because the number of trials is an important measure of the reliability of estimates of variation between trial results (i.e., I^2^). Since we accept the final cumulative I^2^ as representing a good approximation of the ‘truth’, it was important that the number of trials was large enough to make it likely that the final I^2^ had converged and was stable.
The total number of patients surpassed a required information size (i.e., required meta-analysis sample size) based on α = 5% and β = 20% (i.e., 80% power). The required information size used for each meta-analysis was powered to detect a 25% relative risk reduction assuming control group event rate equal to the median of all trials. Calculation of a required information size requires an estimation of heterogeneity [23]. For the purpose of estimating a reasonable required information size (and allowing confidence that our final effect estimate is reliable), we chose to assume a 50% degree of heterogeneity for these calculations. I^2^ is a function of Cochran's Q and Cochran's Q is a function of the sum of squared differences between each trial effect estimate and the meta-analysed effect estimate. Thus, if the meta-analysed effect estimate cannot be considered reliable, I^2^ may not be reliable either. The use a fixed *a priori I^2^* = 50% for the heterogeneity correction is based on a recent comprehensive simulation study, in which we demonstrated that meta-analysed effect estimate is highly unlikely to be over- or underestimated after the cumulative number of patients have surpassed a 50% heterogeneity corrected information size [24].
The disease of interest was a common disease. We employed this criterion because most interventions for common diseases yield intervention effects close to a 25% relative risk reduction or smaller, thus giving credence to our considerations for the required information size (above criterion).

From the pool of 920 meta-analyses, 18 meta-analyses were originally eligible for our analysis, and after further considerations 16 studies were included. Post hoc, we elected to exclude two meta-analyses. These two meta-analyses each included two significantly different subgroups where all or the majority of trials in the second subgroup had been conducted after the trials in the first subgroup. We therefore did not find it appropriate to assess convergence of *I^2^* in this meta-analysis. Table 1 presents the characteristics of the 16 included meta-analyses.

**Table 1:** Characteristics of the 16 included meta-analyses.

Disease/population	Outcome	Experimental intervention	Control Intervention	Period	Cumulative (final) statistics			
					Trials	Events	Patients	*I* ^2^
(1) Colon Cancer	Death	Adjuvant therapy for completely resected stage II cancer	No adjuvant therapy	1987–2007	44	3402	17805	0%
(2) Need for perioperative allogenic blood transfusion	Exposure to allogenic blood	Aprotinin	Blood transfusion & blood loss	1987–2006	96	5348	10144	68%
(3) Bacterial infections in afebrile neutropenic patients following chemotherapy	Febrile patients/episodes	Antibiotic prophylactic drugs	Placebo/no intervention	1973–2005	46	3201	6023	74%
(4) Fever following cesarean section	Fever	Antibiotic prophylaxis	Control	1971–2001	45	1504	7180	49%
(5) Postoperative infection after appendectomy	Wound infection	Antibiotics	Placebo	1986–1995	70	919	8812	26%
(6) Pre-eclampsia and its complications	Gestational hypertension	Antiplatelet agents	Placebo/No antiplatelet agents	1985–2004	33	2080	20701	48%
(7) Need for perioperative allogenic blood transfusion	Exposure to allogenic blood	Cell salvage	Blood transfusion & blood loss	1979–2003	46	1808	3857	77%
(8) Smokers	Smoking cessation at 6+ months follow-up	Nicotine replacement therapy (any type)	Placebo/No therapy control	1979–2007	111	5962	43040	23%
(9) Smokers	Smoking cessation at longest follow-up	Nursing interventions	Control	1987–2005	31	1977	15205	54%
(10) Colorectal cancer	Recurrence of cancer	Perioperative blood transfusion	No intervention	1985–2001	36	4026	12127	59%
(11) Chronic hepatitis C	Sustained virological response	Ribavirin plus interferon	Interferon	1995–2004	54	6126	8354	80%
(12) Rapid sequence induction intubation	Intubation condition	Rucoronium	Succinylcholine	1992–2006	37	1948	2690	55%
(13) Non-small cell lung cancer in patients with advanced disease	Response to treatment	Double agent regimens	Single agent regimen	1984–2003	33	1410	7175	53%
(14) Metastatic breast cancer	Response to treatment	Single agent	Combination chemotherapy	1975–2003	38	2380	6184	75%
(15) Postoperative pain in adults	>50% pain relief over 4 to 6 hours	Single dose oral paracetamol	Placebo	1975–2006	56	1969	5762	63%
(16) Pregnant women at labor term	Caesarean section	Vaginal prostaglandin (for induction)	Placebo/No treatment	1979–1997	31	898	6243	0%

### Analysis

For each of the 16 meta-analyses we calculated and plotted the cumulative *I^2^* estimate and associated 95% CI after each year of publication. We accepted the final *I^2^* estimate (i.e., the *I^2^* estimated based on the meta-analysis including all trials) as representing a good approximation of the ‘truth’. First, we assessed the variation of *I^2^* estimates over time by calculating the difference between the maximum and minimum observed *I^2^* estimate over time in each meta-analysis. We refer to this difference as the *fluctuation span of I^2^*. Second, we assessed how many events and trials were required for the cumulative *I^2^* estimate to become stable. We defined the considered *I^2^* estimates moderately and highly stable from the points where the cumulative *I^2^* estimate came within a +/−20% and a +/−10% absolute distance of the final cumulative *I^2^* estimate and stayed within this distance. Third, we recorded the cumulative number of trials and events where the 95% CIs temporarily did include the final *I^2^* estimate. At these time points, we assessed how far the closest CI limit was to the final *I^2^* estimate. That is, if the final *I^2^* estimate was above the temporary 95%CI, we calculated the distance between the upper CI limit and the final *I^2^* estimate, and vice versa if the final *I^2^* estimate was below the 95% CI.

## Results

**Figure 1:**
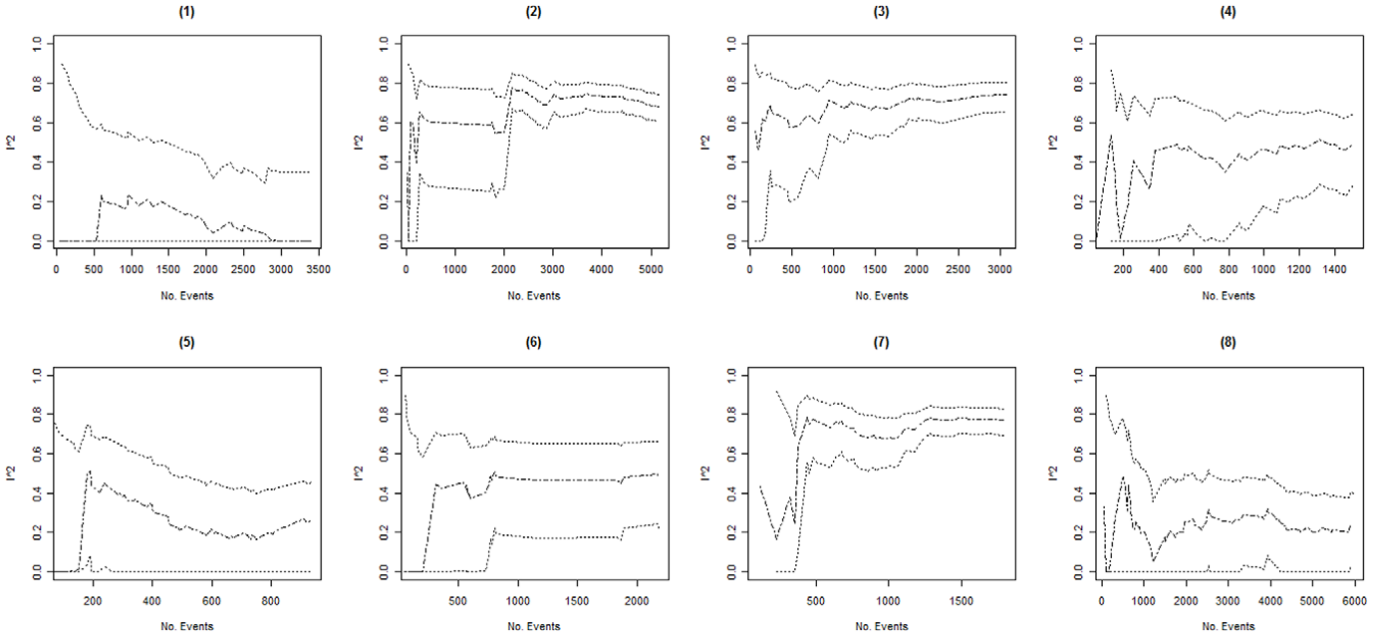
Presents the evolution of the cumulative *I^2^* estimates and their associated 95% confidence intervals (CIs) over the accumulation of events in meta-analyses (1) to (8). The cumulative *I^2^* are represented by the dot-dashed line ( ), and their associated cumulative 95% CIs are represented by the dotted lines ( ).

**Figure 2:**
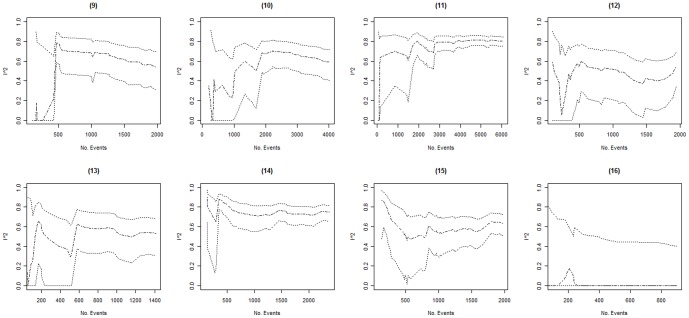
Presents the evolution of the cumulative *I^2^* estimates and their associated 95% confidence intervals (CIs) over the accumulation of events in meta-analyses (9) to (16). The cumulative *I^2^* are represented by the dot-dashed line ( ), and their associated cumulative 95% CIs are represented by the dotted lines ( ).

**Figure 3:**
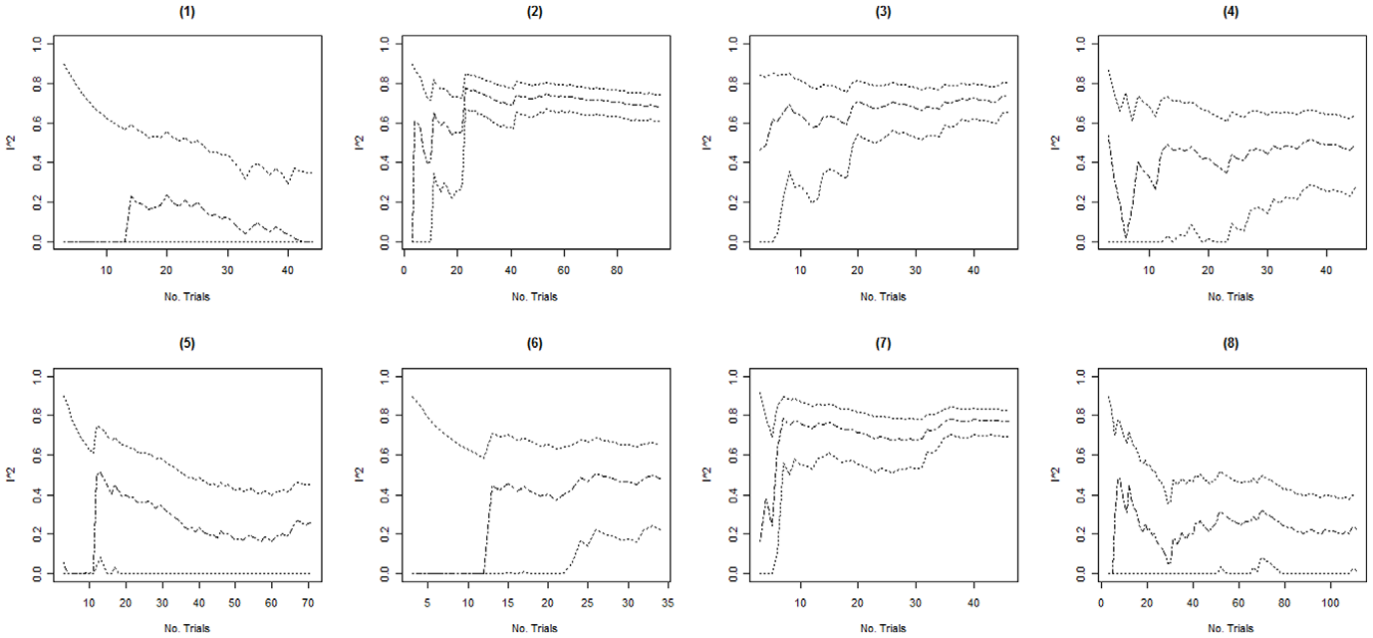
Presents the evolution of the cumulative *I^2^* estimates and their associated 95% confidence intervals (CIs) over the accumulation of trials in meta-analyses (1) to (8). The cumulative *I^2^* are represented by the dot-dashed line ( ), and their associated cumulative 95% CIs are represented by the dotted lines ( ).

**Figure 4:**
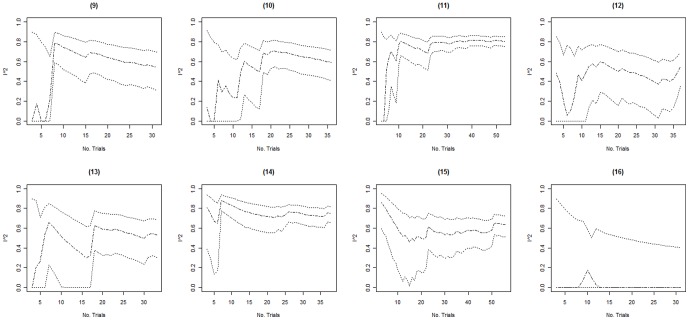
Presents the evolution of the cumulative *I^2^* estimates and their associated 95% confidence intervals (CIs) over the accumulation of trials in meta-analyses (9) to (16). The cumulative *I^2^* are represented by the dot-dashed line ( ), and their associated cumulative 95% CIs are represented by the dotted lines ( ).

Columns 2–4 in table 2 present the minimum, the maximum, and the fluctuation span of *I^2^* values observed over time in each of the 16 included meta-analyses. The median, minimum and maximum fluctuation span was 47.5%, 15%, and 81%. Ten of the 16 meta-analyses (62.5%) had a fluctuation span larger than 40%. Columns 5–8 in table 2 present the number of trials and events required for the cumulative *I^2^* estimate to become moderately and highly stable. In 3 of the 16 meta-analyses (meta-analyses 14–16) the cumulative *I^2^* estimates were moderately stable throughout the entire meta-analysis. For the remaining 13 meta-analyses, the median (minimum to maximum) number of trials and events required to become moderately stable was 11 (5 to 25) and 467 (138 to 1894) respectively. The median (minimum to maximum) number of trials and events required to become highly stable was 20 (10 to 37) and 958 (257 to 2766). Further, graphical inspection revealed that, except for two meta-analysis (meta-analysis 8 and 10, see figures 1, 2, 3, 4), no major fluctuations occurred after the first point where the cumulative meta-analysis included at least 500 events and 15 trials.

**Table 2:** Fluctuation spans of *I^2^* values and the number of trials and events required to become stabile.

Meta-analysis	Minimum *I^2^* value	Maximum *I^2^* value	Flutuation span of *I^2^*	Number of trials required to become stabile		Number of events required to become stabile	
				Moderately (+/−20%)	Highly (+/−10%)	Moderately (+/−20%)	Highly (+/−10%)
(1)	0%	24%	24%	24	33	1297	2096
(2)	42%	74%	32%	11	35	276	2766
(3)	46%	74%	28%	5	19	149	924
(4)	2%	54%	52%	9	28	287	992
(5)	0%	51%	51%	25	34	335	453
(6)	0%	51%	51%	16	16	562	562
(7)	24%	78%	54%	12	12	597	597
(8)	0%	48%	48%	11	33	610	1509
(9)	0%	77%	77%	11	21	537	1393
(10)	24%	69%	45%	18	18	1894	1894
(11)	0%	81%	81%	6	12	138	1989
(12)	10%	57%	47%	13	37	467	1948
(13)	0%	63%	63%	8	18	199	580
(14)	65%	88%	23%	-	10	-	475
(15)	51%	66%	15%	-	24	-	879
(16)	0%	18%	18%	-	13	-	257

In 3 of the 16 meta-analyses (meta-analyses 7, 9 and 14), the 95% CIs temporarily did not include the final *I^2^* estimate (see figures 1, 2, 3, 4). In meta-analysis 7, the 95% CI at the second publication year was 0–69% and the final *I^2^* estimate was 77%. The cumulative number of events and trials at this point was 349 and 5. In meta-analysis 9, the 95% CI at the fourth publication year was 57–88% and the final *I^2^* estimate was 54%. The cumulative number of events and trials at this point was 177 and 5. In meta-analysis 14, the 95% CI at the third and fourth publication year was 77–94% and 75–93% and the final *I^2^* estimate was 74%. The cumulative number of events and trials was 349 and 7 at the third year of publication and 407 and 8 at the fourth year of publication.

## Discussion

In summary, our findings suggest *I^2^* estimates are likely to incur considerable fluctuations when a meta-analysis includes less than roughly 500 events and less than 15 trials, and that 95% CIs for the *I^2^* estimate provide good coverage over time. All instances where the 95% CI temporarily did not include the final *I^2^* estimate occurred in cumulative meta-analyses with less than 500 events and 10 trials. However, even in the rare cases where the 95% CIs did not include the final *I^2^* estimate, it is unlikely that inferences about the degree of heterogeneity based on the temporary 95% CIs would have differed from inferences based on the final *I^2^* estimate.

Our study offers several strengths. First, it represents the first empirical evaluation of the evolution of *I^2^* estimates and their associated 95% confidence intervals over time. Second, our results provide novel insights on the use of one of the most important inferential measures, *I^2^*, in meta-analytic practice. Third, we selected meta-analyses including a sufficiently large number of trials and patients to help ensure a sufficiently reliable sample.

Our study has a number of limitations. We only evaluated *I^2^* estimates and their associated 95% CIs after each year of publication. Since all of the included meta-analyses included more than 1 trial for some of the years, it is possible that the *I^2^* estimates in some of the 16 meta-analyses may have become stable with a smaller number of events and trials than indicated in table 2. Some of the number of events and trials required to reach convergence which we present in table 2 may be therefore overestimates. However, a preliminary analysis plotting the *I^2^* estimates by trial (results not shown), where trials were ordered alphabetically (according to first author's last name or trial acronym) did not reveal additional fluctuations compared with the by-publication-year plots.

Only 16 meta-analyses were eligible, having covered a limited spectrum of medical areas. Our findings may therefore not be generalizable to meta-analyses that bear little resemblance to the meta-analyses included in this study. Similarly, we also did not examine meta-analyses published in paper journals. A number of differences between Cochrane meta-analyses and journal based meta-analyses have been documented (e.g., meta-analyses published in paper journals are more likely to present statistically significant findings) [15], [16]. One could therefore speculate that fluctuations in *I^2^* estimates may differ between Cochrane and paper journal meta-analyses. In the second section of this paper (statistical framework and theoretical considerations), we explained that *I^2^* estimates may fluctuate due to lack of power, time-dependent bias, and evolving trial weights and precisions. We did not perform an in-depth assessment of the degrees to which each of these factors caused *I^2^* estimates to fluctuate in the 16 meta-analyses. We believe empirical analysis of a data set obtained through more lenient eligibility criteria (e.g., only insisting on minimum 15 trials), or specifically tailored simulation studies may have cast more light on these issues. While such studies would provided advantages over this study, they would also come with limitations that are not present in the current study. We therefore believe and in-depth evaluation of causes of *I^2^* fluctuations and convergence are best kept separate from this study. Finally, we did not examine if any of the review authors took any precautions about uncertainty associated with *I^2^* estimates (especially in early versions of the systematic reviews where the meta-analysis included less than 500 events and 15 trials). However, given the paucity of methodological literature on the *I^2^* measure just five years ago, it is likely that most Cochrane review authors would have been unaware of the issues related to uncertainty associated with estimating *I^2^*.

The median number of trials in a meta-analysis is 7 in Cochrane reviews and 12 in systematic reviews published in paper journals [15], [16]. With clinical trial sample sizes typically being smaller than 100 [17], [18], it is likely that most published meta-analyses will incur considerable fluctuations (i.e., meta-analyses with less than 500 events and 15 trials). Hence, there is a need for presenting the *I^2^* estimate with its associated 95% CI. As outlines in the statistical framework section, 95% CIs represents the spectrum of results that would include the true underlying value in 95% of all ‘meta-analysis experiments’. As such, clinicians should consider what their interpretation of the degree of heterogeneity would be if the *I^2^* estimate was equal to the lower 95% CI limit, and separately the upper 95% CI limit. This should yield an appropriate spectrum of interpretations and adequately reflect the uncertainty.

Unreliable *I^2^* estimates have potential negative implications for the assessment of reliability of intervention effect estimates. Recent literature as well as the GRADE initiative have promoted the need for assessing intervention effects in relation to the strength of evidence [23]–[28]. One of the factors when considering the overall quality of evidence is the precision of the pooled estimate of effect, which is achieved, in part, through considering the required (or optimal) information size [23]–[28]. However, to carry out such assessments reliably it is necessary to have a good idea of the expected degree of heterogeneity in the meta-analysis, and if this is not possible, one should at least carry out sensitivity assessments based on a plausible spectrum of degrees of heterogeneity. Uninformed use of the current *I^2^* estimate does not provide a solid basis for such assessments, but interpretation of the *I^2^* estimate in relation to the cumulative amount of evidence and the associated 95% CI does.

Previous studies have already identified limitations associated with the *I^2^* measure as well as the uncertainty associated with *I^2^* estimates [7], [8], [11], [12]. Our study adds to the previous literature by introducing temporality. However, as pointed out above, our findings do have limitations and need confirmation in simulation studies and perhaps other empirical studies. Example papers, which put statistical inferences about the degree of heterogeneity in a clinical context, are also required. The latter may be realized if confidence intervals became an integral part of the widely used systematic review software Review Manager as well as other meta-analysis software packages [29].

In conclusion, *I^2^* estimates are likely to fluctuate considerably in meta-analyses with less than roughly 500 events and 15 trials. Confidence intervals for *I^2^* estimates provide good coverage as evidence accumulates, and are thus valuable for reflecting the uncertainty associated with estimating *I^2^*. It is our hope that the next updates of systematic review and meta-analysis software packages, such as Review Manager, will include confidence intervals for the *I^2^* estimate.

## Supporting Information

Appendix S1 **I^2^ confidence intervals.**
(DOCX)
