# Supplementary material for: Evolution of Heterogeneity (I2) Estimates and Their 95% Confidence Intervals in Large Meta-Analyses
Source: PLoS One. 2012 Jul 25;7(7):e39471. doi: 10.1371/journal.pone.0039471 (PMC3405079; doi:10.1371/journal.pone.0039471)
Supplement: Appendix S1 — I2 confidence intervals. (DOCX) [file pone.0039471.s001.docx]

**Appendix**

Let *Q* be Cochran’s homogeneity test statistic and let *k* be the number of trials included in a meta-analysis. The calculation of confidence intervals for *I^2^* using the ‘test based’ methods relies on a transformation of the *I^2^* statistic, *H^2^=1/(1- I^2^)=Q/(k-1)*. The test based method utilizes that the statistic *Z=√(2Q)-√(2k-3)* approximately follows a standard normal distribution. Similarly, if we take the natural logarithm of *Q* we remove much of the skewness of the underlying distribution of *Q*. Because the expectation of *Q* is *k-1*, *(ln(Q)-ln(k-1))/(SE(ln(Q)))* can be assumed to approximately follow a standard normal distribution. Equating Z with this expression and isolating for *SE(ln(Q))* we get

$$SE(\ln(Q))=\frac{\ln(Q)-\ln(k-1)}{\sqrt{2Q}-\sqrt{2k-3}}$$

And since *Q=(k-1)H^2^* and *k* is a constant we have *SE(ln(Q))=2*SE(ln(H)),* and hence

$SE(\ln(H))=\frac{1}{2}\frac{\ln(Q)-\ln(k-1)}{\sqrt{2Q}-\sqrt{2k-3}}$ (1)

One problem with this approach is that the standard error approaches zero as *H* approaches 1. Small values of *H* indicate homogeneity of trial results in which case *Q* is χ^2^ distributed with *k-1* degrees of freedom. In this case we can take an approximate variance of *ln(Q/(k-1))=2*ln(H)* and arrive at

$SE(\ln(H))=\sqrt{\frac{1}{2(k-2)}\left(1-\frac{1}{3(k-1)^{2}}\right)}$ (2)

Higgins et all showed via simulation that formula (1) should be used when Q>k and formula (2) should be used when *Q≤k*. Having estimated the standard error of *ln(H)* one can assume approximate normality and derive approximate 95% confidence intervals for *H*: *exp(ln(H)±1.965*SE(ln(H))).* One can subsequently square the resulting CI limits and transform each of them back to a percentage of heterogeneity, *I^2^*.[2]
